# Actin polymerization is activated by terahertz irradiation

**DOI:** 10.1038/s41598-018-28245-9

**Published:** 2018-07-03

**Authors:** Shota Yamazaki, Masahiko Harata, Toshitaka Idehara, Keiji Konagaya, Ginji Yokoyama, Hiromichi Hoshina, Yuichi Ogawa

**Affiliations:** 1 Laboratory of Molecular Biology, Graduate School of Agricultural Science, Tohoku University, Aramaki Aza Aoba 468-1, Aoba-ku, Sendai 980-0845 Japan; 2 Terahertz Sensing and Imaging Research Team, RIKEN Center for Advanced Photonics, 519-1399 Aramaki-Aoba, Aoba-ku, Sendai, Miyagi 980-0845 Japan; 3 Research Center for Development of Far-Infrared Region, University of Fukui (FIR UF), Bunkyo 3-9-1, Fukui, 910-8507 Japan; 4 Graduate School of Agriculture, Kyoto University, Kitashirakawa-Oiwakecho, Sakyo-ku, Kyoto 606-8205 Japan

## Abstract

Polymerization of monomeric actin into filaments has pivotal roles in cell motility, growth, differentiation, and gene expression. Therefore, techniques of manipulating actin polymerization, including actin-binding chemicals, have been developed for understanding and regulating multiple biological functions. Here, we demonstrate that irradiation with terahertz (THz) waves is a novel method of modulating actin polymerization. When actin polymerization reaction is performed under irradiation with 0.46 THz waves generated by a Gyrotron, actin polymerization was observed to be activated by monitoring the fluorescence of pyrene actin fluorophores. We also observed the number of actin filaments under a fluorescence microscope using the polymerized actin probe SiR-actin. The number of actin filaments was increased by 3.5-fold after THz irradiation for 20 min. When the THz irradiation was applied to a steady-state actin solution, in which elongation and depolymerization of actin filaments were equilibrated, increased actin polymerization was observed, suggesting that the THz irradiation activates actin polymerization, at least in the elongation process. These results suggest that THz waves could be applied for manipulating biomolecules and cells.

## Introduction

The recent development of high-power THz sources enables novel scientific experiments in various fields^1^. In particular, extremely high-power radiation sources, i.e., gyrations for which the frequency is increased to the THz range, can be applied to high-power THz spectroscopy in many research fields, for example, direct measurement of the hyper-fine structure of positronium in elementary particle physics^2^ and enhancement of the sensitivity of NMR spectroscopy using dynamic nuclear polarization (DNP) to analyze the complicated structure of protein molecules in the life sciences^3–5^.

Electromagnetic fields obtained by ultrafast THz pulses produce unexplored non-linear physical phenomena such as molecular orientation^6^, insulator-to-metal transition^7^, and coherent excitation of the vibrational transition^8,9^. However, most of these phenomena are transient, as they are induced by picosecond THz pulses and probed by ultrafast spectroscopy. Changing the material structure permanently, using THz irradiation, has been challenging because of the fast relaxation at room temperature. Recently, Hoshina *et al*.^10^ succeeded in changing the crystallinity of polymer films using irradiation by THz waves during sample formation from solution. Given that the energy of THz photons is quite low compared to that of covalent bonds, molecular damage such as ionization rarely occurs. Therefore, THz wave irradiation can alter the macromolecular structure ‘softly’ just by exciting intermolecular interactions.

THz wave irradiation has attracted keen interest in the biological field. It was previously reported that THz waves do not induce direct chromosomal damage or alter cell cycle kinetics or skin cell proliferation^11–13^. Although these studies indicated the safety of THz irradiation, some reports suggest that high-power THz waves cause adverse effects. Recent reports showed that THz irradiation inhibits cell proliferation and changes the adhesive properties of the nerve cell membrane^14,15^. Changes in transcriptional activation and induction of apoptotic processes in human dermal fibroblasts and Jurkat cells have also been demonstrated^16,17^. Furthermore, irradiation of mouse stem cells with broadband THz pulses altered gene expression in mammalian cells^18^. However, given that the properties of living cells are regulated by various intracellular macromolecules, such as DNA, protein complexes, and cytoskeletal components, the mechanism and exact targets of THz waves are poorly defined.

In this study, we applied 0.46 THz wave pulses to actin solution during polymerization. Actin is one of the most abundant cytoplasmic proteins and has two functional forms: monomeric globular (G)- and polymerized filamentous (F)-actin. Actin filaments form an elaborate network called the cytoskeleton, which plays crucial roles in cell shape, motility, and division^19,20^. In living cells, actin polymerization is regulated by various actin binding proteins (ABPs) and actin-related proteins (Arps) in living cells^21,22^. Importantly, actin polymerization can be reconstituted with purified G-actin^23^, and the polymerization process of purified actin consists of three phases: nucleation, elongation, and steady state. This *in vitro* actin polymerization reaction is suitable for analyzing the influence of THz waves on biomolecules. Because filamentous actin has pivotal roles in the functions of normal and pathological cells, including metastasis of cancer cells, various chemical compounds affecting actin polymerization have been analyzed for research and therapeutic purposes^24,25^. Our finding of the enhancement of actin polymerization by THz irradiation suggests a novel possibility of artificial manipulation of biomolecules and living cells using THz waves.

## Results

### THz wave irradiation of actin solution

**Figure 1:**
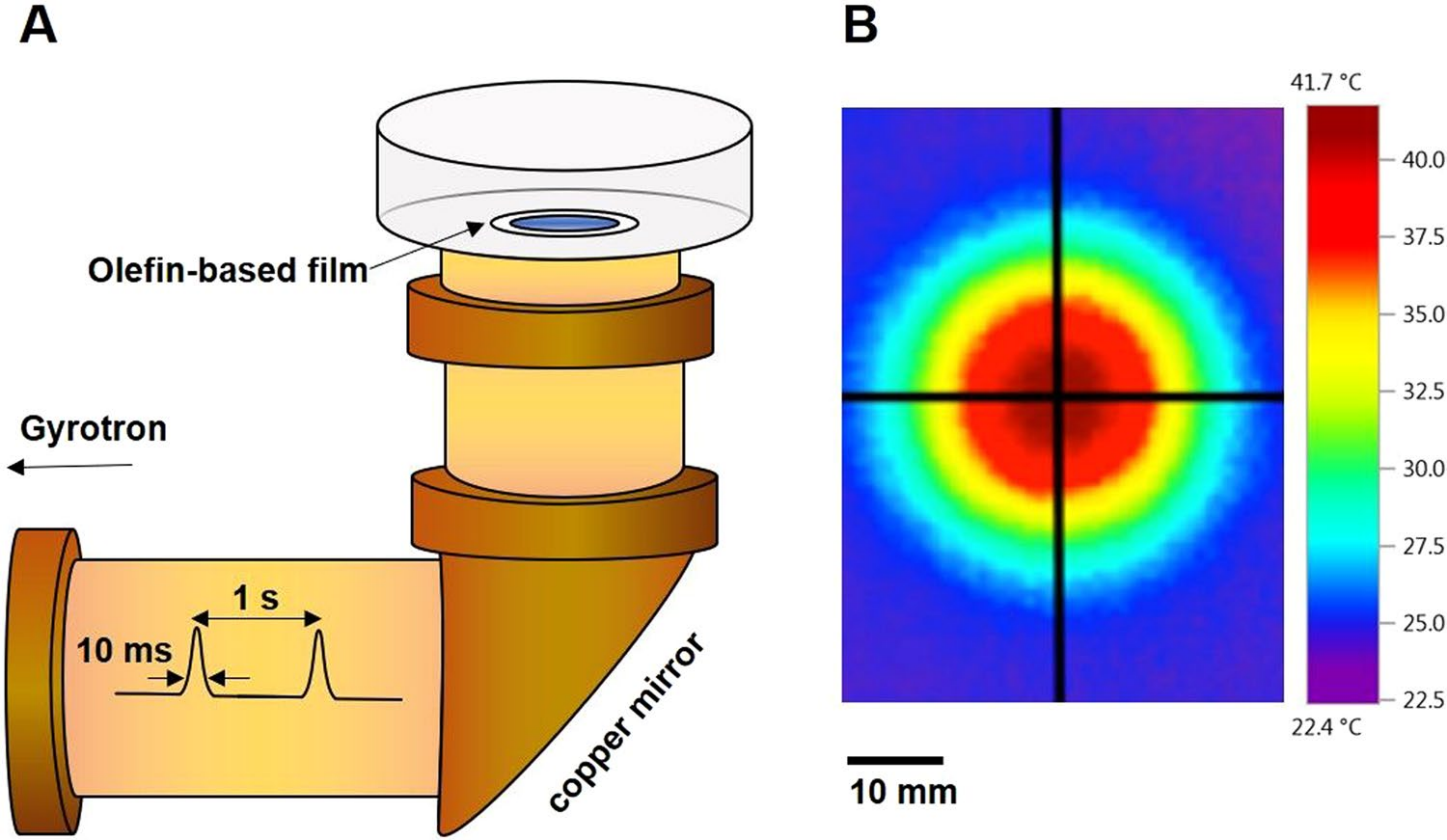
Schematic representation of experimental conditions. (**A**) Irradiation with THz waves generated by a Gyrotron in the pyrene actin solution. The solution was placed on an olefin-based film over the waveguide and subjected to irradiation at 25 °C. (**B**) The beam profile of the THz wave output measured by thermography.

The 0.46 THz wave generated by a Gyrotron (FU CW VIB, developed at Fukui University; also known as FU CW GOIII developed at Osaka University) was applied to actin solutions and polymerization was observed. Actin polymerization was initiated by adding the F-buffer to G-actin solution and proceeded at 25 °C. Because THz waves do not penetrate glass, the actin solutions were set on an olefin-based film dish and the dice was placed over the waveguide from which the THz wave was vertically irradiated (Fig. 1A). The beam profile of the THz output is shown in Fig. 1B. The THz output formed a Gaussian shape with a full-width half maximum of 30 mm.

### Monitoring of pyrene actin polymerization with THz irradiation

**Figure 2:**
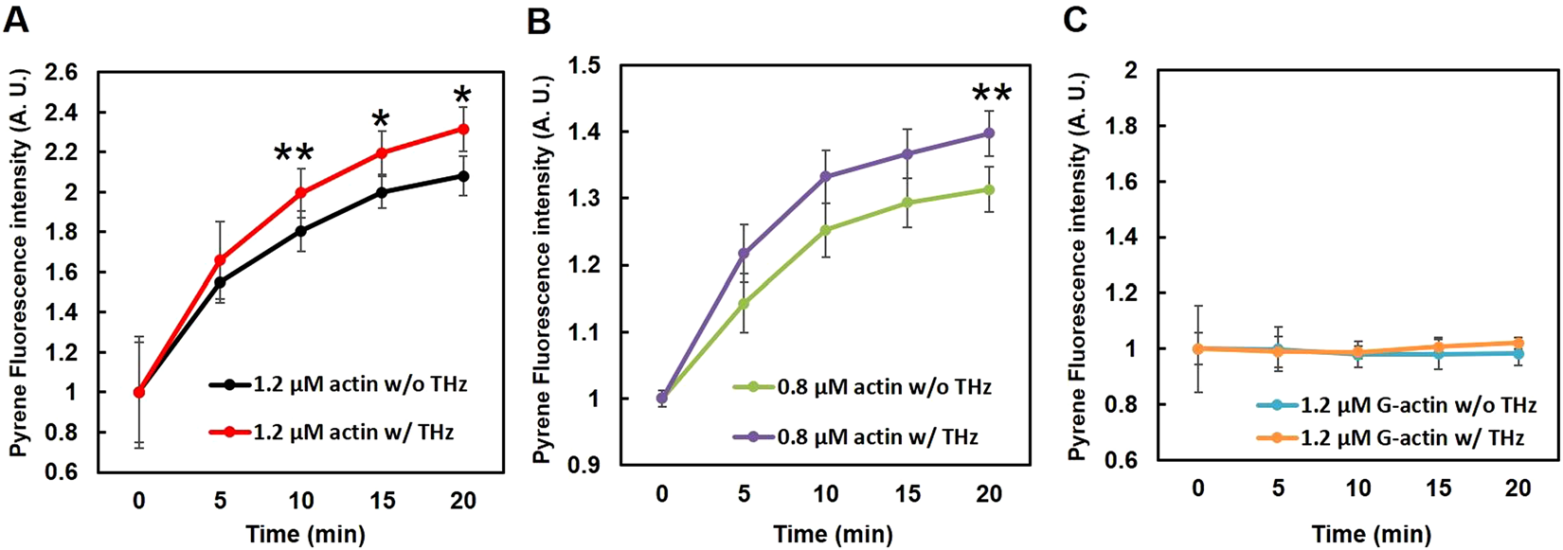
Monitoring of actin polymerization using pyrene actin. (**A** and **B**) Pyrene-labeled actin solution was polymerized by adding F-actin buffer at the time point of 0, and the increase in the fluorescence of pyrene was observed by using GloMax-20/20 with Luminometer Fluorescent Modules UV (EX: 365–395 nm, EM: 440–470 nm) with or without irradiation with THz waves (w/THz or w/o THz, respectively). The fluorescent signal was measured every 5 min for 20 min at 25 °C. In panel C, the fluorescence was monitored without adding the F-actin buffer. The initial G-actin concentration was 1.2 μM (**A** and **C**), or 0.8 μM (**B**). The relative fluorescence of pyrene at 0 min was defined as 1.0. Data shown are the mean ± SD of three independent experiments. *P < 0.05, **P < 0.01.

Actin polymerization is generally quantified by measuring the fluorescence of pyrene fluorophores introduced into the actin molecule (pyrene actin). Upon actin polymerization, the fluorescence of pyrene actin is increased^23^. In this study, we measured the fluorescence of the pyrene actin solution in the dish by manually transferring the solution to the cuvette of a luminometer (EX: 365–395 nm, EM: 440–470 nm) for 20 min with 5-min intervals. This reaction represents the elongation phase of actin polymerization. We measured the elongation of actin polymerization under two different concentrations (1.2 μM and 0.8 μM) with or without irradiation with the 0.46 THz wave (Figs 2 and S1). At the concentration of 1.2 µM, the increase in fluorescence from pyrene actin was significantly enhanced by irradiation with the THz wave at 10, 15, and 20 min (Fig. 2A). At a lower actin concentration (0.8 µM), the effect of the THz radiation was less obvious, and a significant enhancement of the fluorescence was observed at 20 min (Fig. 2B). When actin polymerization was continued after stopping the THz irradiation at 20 min, there was no difference in the fluorescence between control and THz-irradiated samples at a steady state (Suppl. Fig. S2). To test whether the THz wave directly affect the fluorescence of pyrene fluorochrome itself, the G-actin solution was irradiated with the THz wave without initiating polymerization. In this case, the THz wave did not affect the fluorescence of pyrene actin (Fig. 2C). These results suggest that the application of the THz wave to actin activates its polymerization.

### Observation of silicon-rhodamine-stained actin filaments under fluorescent microscope

**Figure 3:**
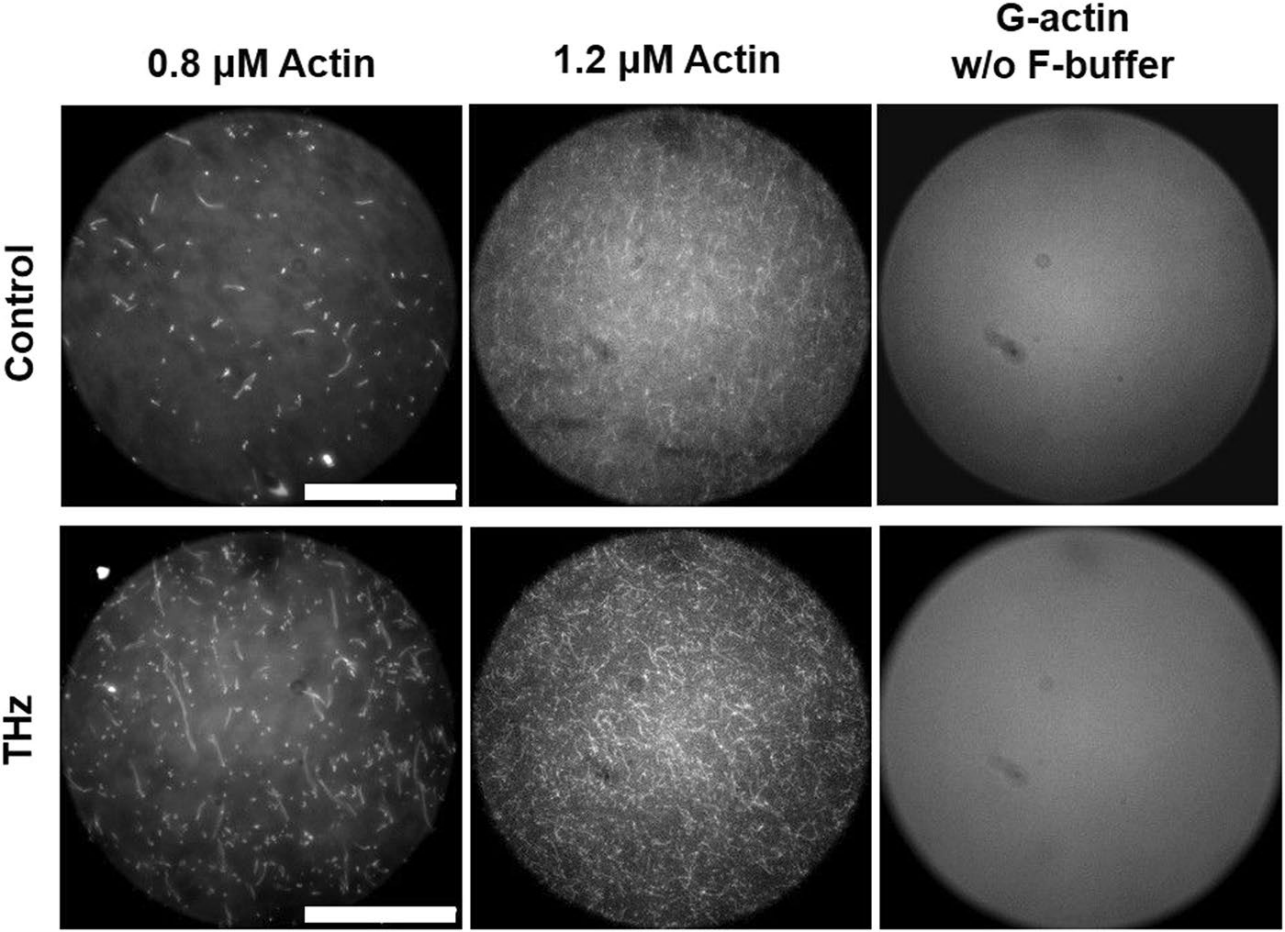
Observation of filamentous actin using the F-actin probe SiR-actin and a fluorescence microscope. G-actin solutions (1.2 μM and 0.8 μM) were polymerized by adding F-actin buffer with or without the THz irradiation (THz or Control, respectively) for 20 min. Then, actin filaments were stained with SiR-actin and observed under a fluorescence microscope. As a negative control, the G-actin solution without initiation of polymerization was stained with SiR-actin and subjected to microscope observation. Bar: 50 μm. More details are described in the Materials and Methods.

To confirm the activation of actin polymerization by THz irradiation, we observed actin filaments under a microscope after THz irradiation (Fig. 3). The structure of actin filaments is generally observed using electron microscopy^26^ or by total reflection fluorescence microscopy^27^. As a simpler method to observe actin filaments, we developed a method using an F-actin probe, silicon-rhodamine (SiR)-actin^28^. The fluorescence of SiR-actin increases by up to 100-fold when it bonds to actin filaments, and therefore it was expected that actin filaments bound with SiR-actin would be observed under widefield microscopy. When an actin solution was subjected to polymerization for 20 min and stained with SiR-actin, we observed filamentous structures with a length of >1 µm under microscopy (Fig. 3, control). These filamentous structures were not observed in the actin solution without initiation of the polymerization reaction (Fig. 3, the right panels), indicating that actin filaments were observed with this method. When the THz wave was irradiated during the polymerization reaction as shown in Fig. 2, similar actin filaments were observed, but the number of filaments was increased in both 1.2 μM and 0.8 μM actin solutions (Fig. 3, THz). This result confirms that THz irradiation activates actin polymerization, but did not induce protein denaturation or aggregation.

### THz irradiation activated actin polymerization

**Figure 4:**
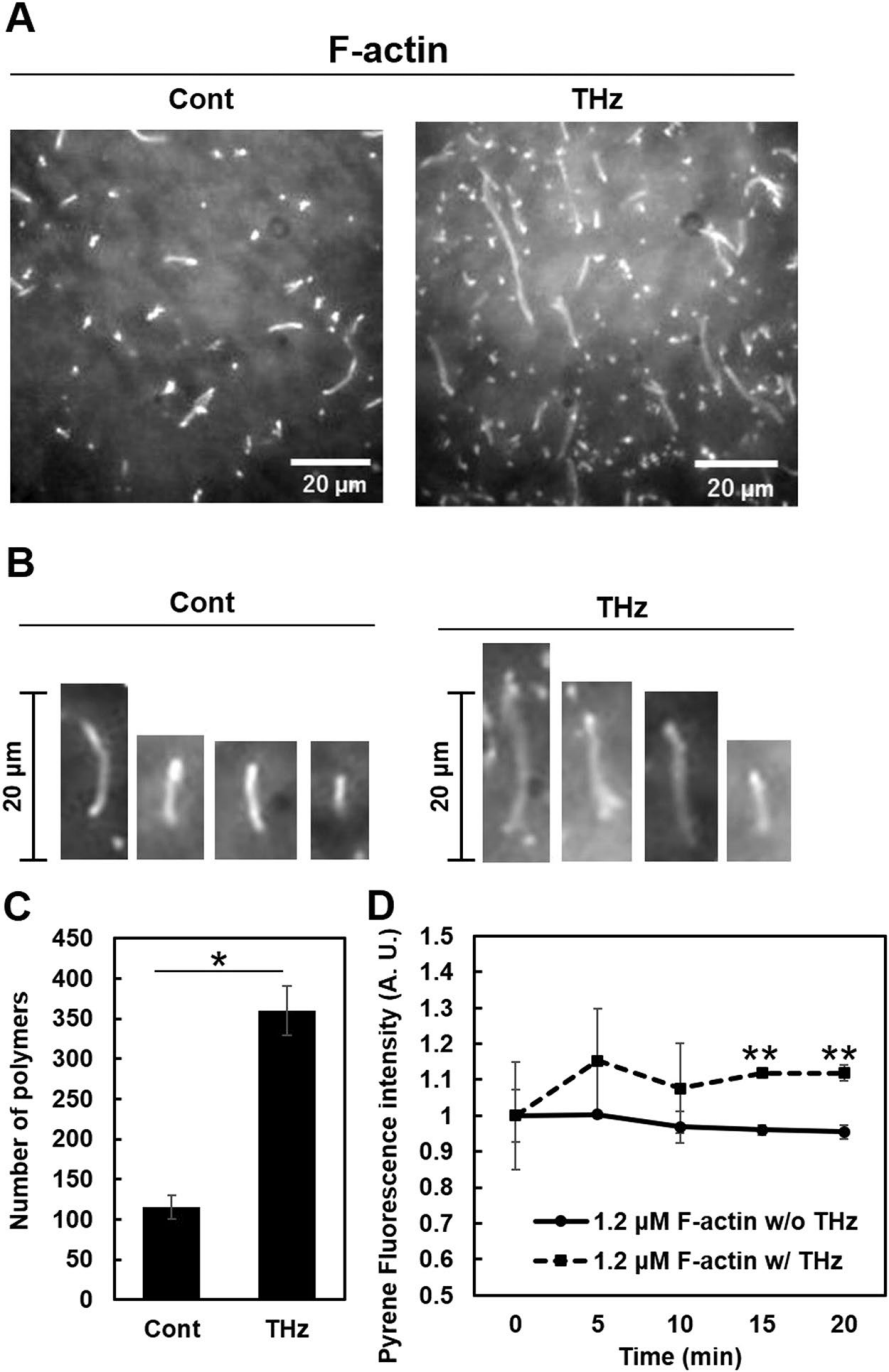
Effect of THz irradiation on actin filaments. G-actin solution (0.8 μM) was polymerized by adding F-actin buffer with or without THz irradiation (THz or Cont, respectively) for 20 min. (**A**) Magnified images of actin filaments with or without THz irradiation. Bar: 20 μm. (**B**) Comparison of the morphology of actin filaments between the control and the THz-irradiated samples. Bar: 20 μm. (**C**) Comparison of the number of actin filaments between the control and THz-irradiated samples. Relative numbers are shown with the control sample as 1.0. Date shown are the mean ± SD of three independent experiments. More than 100 actin filaments were counted in each of the experiments. *P < 0.05. (**D**) Pyrene actin filaments were formed for 1 h and then fluorescence was measured at each time with or without THz irradiation (w/THz or w/o THz, respectively). The relative fluorescence of pyrene at 0 min was defined as 1.0. Data shown are the mean ± SD of three independent experiments. **P < 0.01.

By using magnified microscopy images, actin polymers with a length of >1 µm were counted, and the effects of the THz irradiation were analyzed (Fig. 4A and B). No morphological difference such as branching or molecular aggregation was observed after THz irradiation (Fig. 4A and B, THz). The total number of actin filaments was increased 3.5-fold by THz irradiation (Fig. 4C).

Actin polymerization, which is activated by THz irradiation, consists of two processes: the nucleation process of the actin monomer in the beginning and the subsequent elongation of actin filaments. At present, our experimental system does not have sufficient resolution for observing the nucleation process; the measurement of pyrene actin has 5-min intervals and the microscopy imaging is available only for filaments with a length of >1 µm. To test the effect of THz irradiation on the elongation of actin filaments, 1.2 μM actin solution was subjected to the polymerization reaction for 1 h without THz irradiation and induced into the steady state, in which elongation and depolymerization of actin filaments were equilibrated. Indeed, the fluorescence of pyrene actin did not increase without THz irradiation in the steady-state actin solution (Fig. 4D, control). However, the application of THz irradiation to the steady state of actin filaments caused an additional increase in the fluorescence of pyrene actin (Fig. 4D, THz), supporting the possibility that THz irradiation activates actin polymerization, at least in the elongation phase.

## Discussion

In this study, we demonstrated that THz irradiation activates the elongation phase of actin polymerization (Fig. 4). Because polymer formation of chemical and biological molecules is mostly sensitive to temperature, the simplest explanation for the enhancement of actin polymerization might be transient increase of temperature due to the absorption of THz irradiation by water molecules. The upper limit of the temperature increase during the irradiation can be estimated by assuming an adiabatic model, i.e., where all of the THz energy is converted to thermal energy. Owing to the high absorbance of liquid water (160 cm^−1^ at 21 °C, 0.47 THz)^29^, more than 97% of THz photon energy is absorbed within a 100-μm-thick sample. A single THz pulse at conditions of 6 mJ/cm^2^ and 10 ms pulse duration increases the temperature at the surface of the sample by approximately 0.5 °C, and the subsequent thermal diffusion decreases the temperature immediately. However, when we checked the temperature increase in the sample during THz irradiation, no change was observed, indicating that the average temperature increase was less than 0.1 °C. Moreover, actin polymerization was not activated by increasing temperature over our experimental conditions (Suppl. Fig. S3), which is consistent with a previous report^30^. Therefore, it is unlikely that a temperature shift by THz irradiation caused the activation of actin polymerization.

Alternatively, THz energy may directly excite the protein’s dynamic motion. Given that THz frequency corresponds to intermolecular motions such as vibration, liberation, and rotational relaxation, irradiation with intense THz waves may induce intermolecular conformational changes^31^. In the case of the actin solution, intermolecular conformations of actin polymers and actin-water complexes can be changed by intense THz waves. A single actin molecule is formed by two major domains, which are linked by a hinge domain. The structural difference between G- and F-actin is the relative rotation of the two major domains via the hinge domain by approximately 20°, which gives the F-actin subunit a flat conformation^32^. More importantly, this conformation change plays an essential role in actin polymerization. Therefore, it seems likely that THz irradiation excites the inter-domain flipping motion of actin molecules, leading to the activation of actin polymerization. However, it is difficult to conclude the exact influencing mechanism of THz irradiation in our work. Therefore, future studies will focus on investigating the details concerning the structure of actin under THz irradiation by changing parameters such as wavelength or intensity and by using actin mutants in which the characteristics of actin are altered. In addition, since the pyrene actin fraction used in this study would contain polymerization nuclei, application of a highly purified G-actin fraction and improved measurement equipment to our work could provide further information on the molecular mechanisms underlying the effects of THz waves on actin polymerization. Moreover, development of microscopy systems for imaging actin filaments under THz irradiation would contribute to analyses of the effect of THz waves on actin in cells.

It is known that irradiation with THz waves does not cause cellular or DNA damage. Therefore, THz irradiation should be a safe and novel technology for regulating the dynamics of actin polymerization and depolymerization in living cells. The actin dynamics in the cytoplasm play pivotal roles in the proliferation and the motility of cells. In addition, actin dynamics in the cell nucleus are required for transcriptional regulation^33–35^. Indeed, some actin-binding chemical compounds are expected to act as anti-cancer drugs because actin dynamics contribute to metastasis of cancer cells. As it is difficult to control the delivery and clearance of these chemicals in target cells, physical irradiation by THz waves could provide an advantage. Our findings suggest that THz waves could be applicable for artificial manipulation of these cellular functions through modulation of actin dynamics.

## Materials and Methods

### Exposure setup

To generate THz pulses, we used a Gyrotron FU CW GO-III^3^ and designed an apparatus which can expose samples to 0.46 THz radiation with a power density of 5.7 mJ/cm^2^. A schematic representation of the device is shown in Fig. 1. The THz-Gyrotron outputted macro-pulses with a 1-Hz repetition rate, each pulse being 10 ms long. The temperature increase of the sample during THz irradiation was measured using a K-type thermocouple. Data shown are the mean ± s.e.m. of three independent experiments. Pyrene actin solution was placed on an olefin-based film dish (0.1-mm thickness and 14-mm width) (Matsunami Glass), which absorbed approximately 10% of the THz radiation (data not shown).

### Actin polymerization assay

The actin polymerization Biochem Kit (Cytoskeleton, Inc) was used to confirm the effect of actin polymerization; this kit is based on enhancement of the fluorescence of pyrene-labeled G-actin that occurs during polymerization, in particular the increase in fluorescence emission at 395–440 nm (excitation wavelength, 340–380 nm). Pyrene fluorescence was measured by using GloMax-20/20 with Luminometer Fluorescent Modules UV (365–395 nm EX, 440–470 nm EM). To prepare pyrene G-actin, rabbit muscle actin solution containing >99% pyrene-labeled actin was dissolved in G-buffer (5 mM Tris-HCl pH 8.5, 0.2 mM CaCl_2_) at 0.4 mg/ml and placed on ice for 1 h. Depolymerized actin solution was centrifuged at 18,400 × g at 4 °C for 30 min and the supernatant was used as G-actin solution. Polymerization was initiated by adding 10 × F-buffer (500 mM KCl, 20 mM MgCl_2_, 50 mM guanidine carbonate, and 10 mM ATP) for a final reaction volume of 150 µl. The actin reaction solution was placed on an olefin-based film dish (0.1-mm thickness and 14-mm width) (Matsunami Glass), and the dish was placed over the waveguide from which the THz wave was vertically irradiated as shown in Fig. 1B.

### Imaging of actin filaments under microscopy

After THz irradiation, 1 µl of actin solution was collected from the film dish and mixed with 9 µl of 5.6 µg/ml SiR-actin (Cytoskeleton, Inc.), and then 1 µl of the mixture was immediately mixed with 3 µl of Vectashield Mounting Medium (Vector Laboratories) and mounted on a slide glass. Prepared actin solutions were observed using IX83 fluorescence microscopy (Olympus). Images were captured with an ORCA-Flash 4.0 LT PLUS Digital CMOS camera (Model C11440-42U30, Hamamatsu). Numbers of actin filaments were measured from obtained images using Image J software.

## Electronic supplementary material

SUPPLEMENTARY INFO
