# Supplementary material for: Actin polymerization is activated by terahertz irradiation
Source: Sci Rep. 2018 Jul 3;8:9990. doi: 10.1038/s41598-018-28245-9 (PMC6030223; doi:10.1038/s41598-018-28245-9)
Supplement: Supplementary file 1 — SUPPLEMENTARY INFO [file 41598_2018_28245_MOESM1_ESM.pdf]

Title

Actin polymerization is activated by terahertz irradiation

Authors

Shota Yamazaki[1,2,*], Masahiko Harata[1,*], Toshitaka Idehara[3], Keiji Konagaya[4], Ginji Yokoyama[1], Hiromichi Hoshina[2], Yuichi Ogawa[4,*].

Author information

[1]Laboratory of Molecular Biology, Graduate School of Agricultural Science, Tohoku University, Aramaki Aza Aoba 468-1, Aoba-ku, Sendai 980-0845, Japan.

[2]Terahertz Sensing and Imaging Research Team, RIKEN Center for Advanced Photonics, 519-1399 Aramaki-Aoba, Aoba-ku, Sendai, Miyagi 980-0845, Japan.

[3]Research Center for Development of Far-Infrared Region, University of Fukui (FIR UF), Bunkyo 3-9-1, Fukui 910-8507, Japan.

[4]Graduate School of Agriculture, Kyoto University, Kitashirakawa-Oiwakecho, Sakyo-ku, Kyoto 606-8205, Japan.

*Corresponding authors: correspondence should be addressed to shota.yamazaki.fc@riken.jp, masahiko.harata.b6@tohoku.ac.jp, or ogawayu@kais.kyoto-u.ac.jp

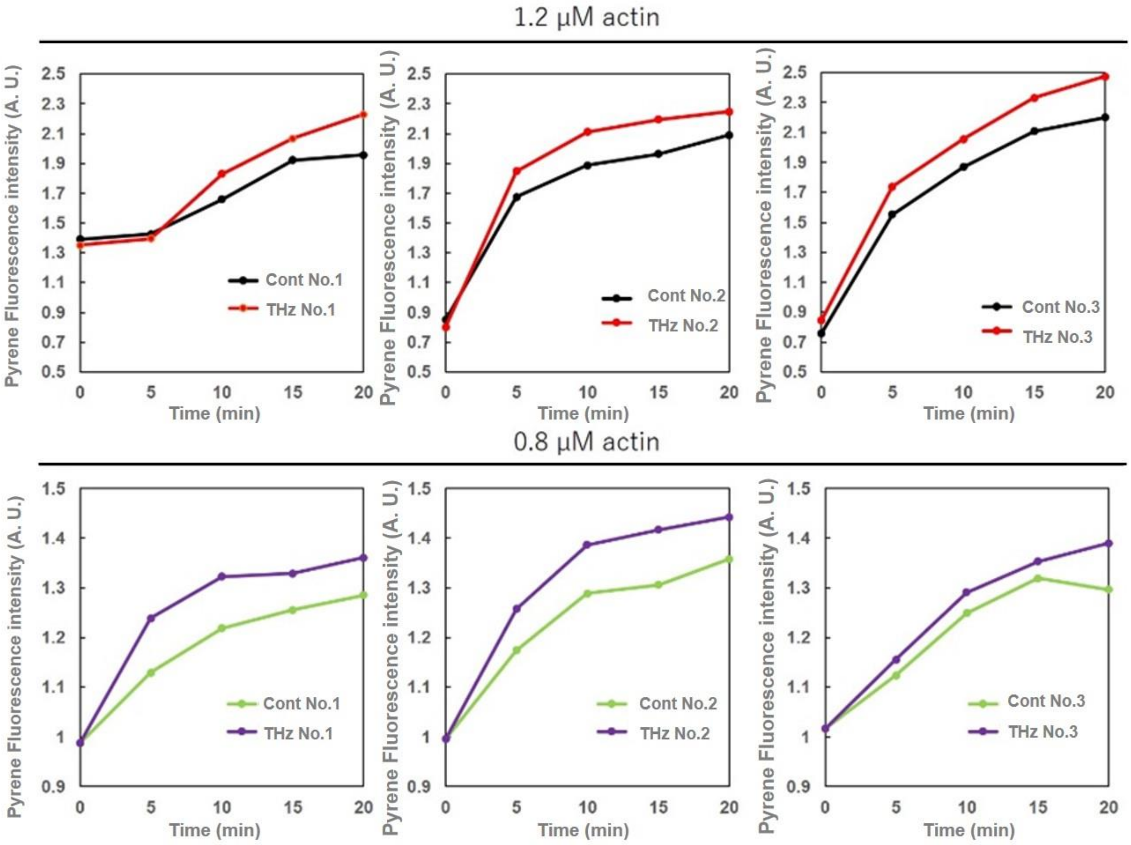

Suppl. Fig. S1. Individual results of actin polymerization reactions shown in Fig. 2A and B.

Pyrene-labelled actin solution was polymerized by adding F-actin buffer at the time point of 0, and the increase in the fluorescence of pyrene was observed with or without irradiation with THz waves. The fluorescent signal was measured every 5 minutes for 20 min at 25°C. Actin concentration is 1.2 µM or 0.8µM.

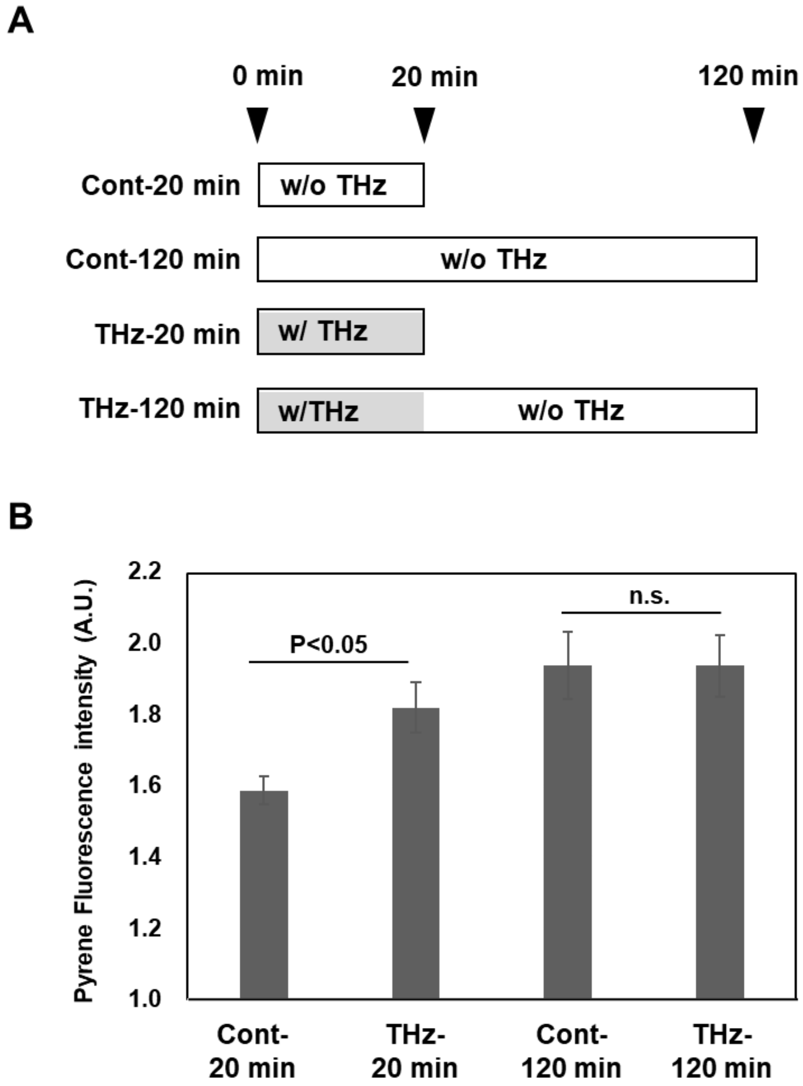

Suppl. Fig. S2. Fluorescence of a steady-state pyrene actin solution after THz irradiation. (A) Schematic representation of the measurement of a steady-state pyrene actin solution after 20 min irradiation of the THz wave. (B) Pyrene-labelled actin solution (2.6 μM) was polymerized by the addition of F-actin buffer to G-actin solution at time point 0, and the increase in the fluorescence of pyrene was observed using GloMax-20/20 with Luminometer Fluorescent Modules UV (EX: 365–395 nm, EM: 440–470 nm). The G-actin solution was prepared as in Fig. 2, except that the centrifugation prior to the addition of F-actin buffer was omitted. The fluorescence signal was measured at 20 min for cont-20 min and THz-20 min samples, and at 120 min for cont-120 min and THz-120 min samples. The relative fluorescence of pyrene at 0 min was defined as 1.0. Data shown are means ± SD of three independent experiments.

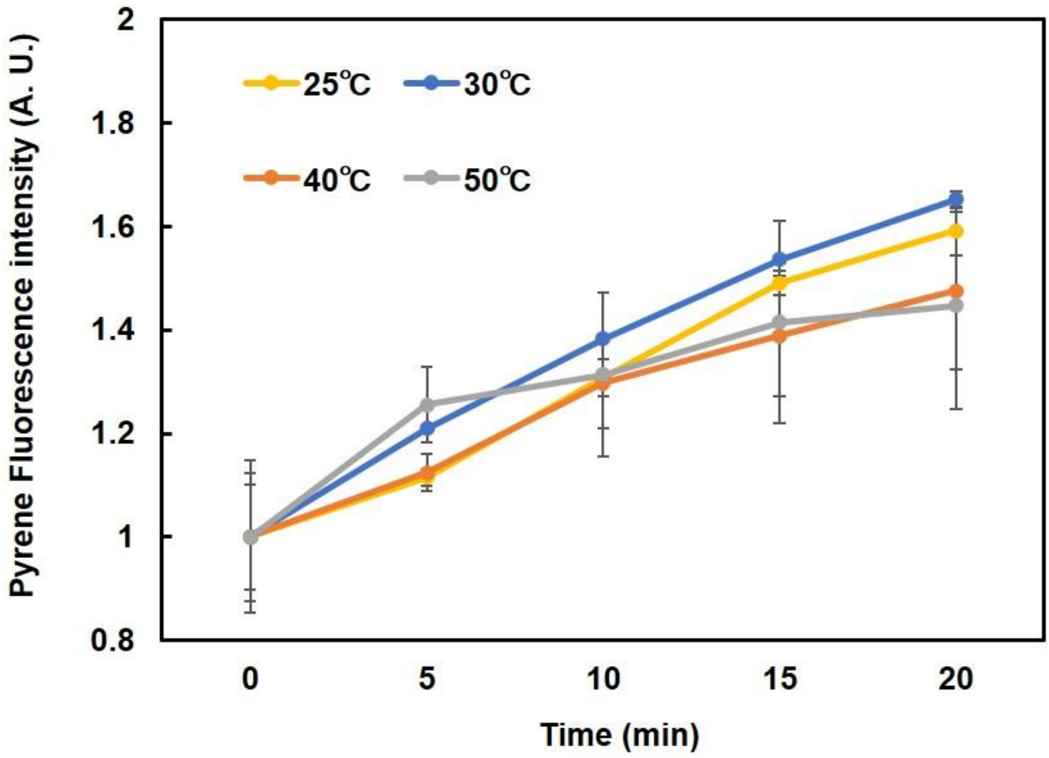

Suppl. Fig. S3. Monitoring of actin polymerization at several temperature conditions. Pyrene-labelled actin solution was polymerized by addition of F-actin buffer at time point 0, and the increase in the fluorescence of pyrene was observed by using GloMax-20/20 with Luminometer Fluorescent Modules UV (EX: 365-395 nm, EM: 440-470 nm). The fluorescence signal was measured every 5 minutes for 20 min at 25, 30, 40, and 50°C. The relative fluorescence of pyrene at 0 minute was defined as 1.0. Data shown are the mean ± SD of three independent experiments.
